# Serum growth differentiation factor-15 and non-esterified fatty acid levels in patients with coronary artery disease and hyperuricemia

**DOI:** 10.1186/s12944-023-01792-5

**Published:** 2023-03-02

**Authors:** Jingru Cheng, Yongnan Lyu, Yufeng Mei, Qian Chen, Hang Liu, Yan Li

**Affiliations:** 1 Department of Clinical Laboratory,institute of translational medicine, Renmin Hospital of Wuhan University, Wuhan, China; 2 Department of Cardiology, Renmin Hospital of Wuhan University, Wuhan, China

**Keywords:** Hyperuricemia, CAD, GDF-15, NEFA

## Abstract

**Background:**

High serum NEFA and GDF-15 are risk factors for CAD and have been linked to detrimental cardiovascular events. It has been hypothesized that hyperuricemia causes CAD via the oxidative metabolism and inflammation. The current study sought to clarify the relationship between serum GDF-15/NEFA and CAD in individuals with hyperuricemia.

**Methods:**

Blood samples collected from 350 male patients with hyperuricemia(191 patients without CAD and 159 patients with CAD, serum UA > 420 μmol/L) to measure serum GDF-15 and NEFA concentrations with baseline parameters.

**Results:**

Serum circulating GDF-15 concentrations(pg/dL) [8.48(6.67,12.73)] and NEFA levels(mmol/L) [0.45(0.32,0.60)] were higher in hyperuricemia patients with CAD. Logistic regression analysis revealed that the OR (95% CI) for CAD were 10.476 (4.158, 26.391) and 11.244 (4.740, 26.669) in quartile 4 (highest) respectively. The AUC of the combined serum GDF-15 and NEFA was 0.813 (0.767,0.858) as a predictor of whether CAD occurred in male with hyperuricemia.

**Conclusions:**

Circulating GDF-15 and NEFA levels correlated positively with CAD in male patients with hyperuricemia and measurements may be a useful clinical adjunct.

## Introduction

Hyperuricemia (HUA) is characterized by an excess of uric acid (UA) synthesis over excretion. The condition causes gout and kidney stones and its prevalence is on the increase [1, 2]. However, HUA has also been linked to increased risk of mortality and morbidity from coronary artery disease (CAD) [3]. Molecular signals produced by oxidative stress, insulin resistance, endothelial dysfunction or the inflammatory response allow HUA to stimulate the onset and progression of CAD [4]. Thus, the risk of CAD in individuals with HUA has been the focus of recent research.

Circulating growth differentiation factor 15 (GDF-15), expressed in liver, kidney, intestine and placenta abundantly, is a biological marker of negative prognosis in cardiovascular disease [5, 6]. The precise cause of elevated serum GDF-15 in individuals with cardiovascular events is uncertain but possible explanations include heart failure, ischemia–reperfusion and atherosclerosis-induced cardiovascular injury [6–8]. It has been observed that GDF-15, which is expressed in endothelial cells, cardiomyocytes, and adipocytes, is connected to ventricular remodeling and decreased ejection fraction [9]. Due to paracrine/autocrine signaling, it is upregulated by several types of cardiac stress in addition to inflammation [10, 11]. By preventing CCR2-mediated chemotaxis and controlling cell death, GDF-15 deletion has a positive effect on both early and late stages of atherosclerosis [12]. The atherosclerosis which contributes to CAD development is characterized by aberrant lipid accumulation, inflammation and smooth muscle cell proliferation and UA may promote atherosclerosis by interfering with lipid metabolism [13, 14]. There has been much investigation of lipid profiles in the context of cardiovascular disease and excessive non-esterified fatty acid (NEFA) is a hallmark of aberrant glucose and lipid metabolism and an unhealthy lifestyle. Elevated NEFA is linked to obesity and diabetes but is also an independent risk factor for CAD [15].

However, any link between serum GDF-15 or NEFA and CAD in men with HUA requires clarification.

## Methods

### Study population

All participants in this study were male HUA patients admitted to the Clinical Endocrinology Department and Cardiovascular Medicine Department, Renmin Hospital of Wuhan University from February 2021 to November 2021. According to inclusion and exclusion criteria, a final total of 350 participants were enrolled. Based on the results of coronary angiography, they were separated into two groups: HUA without CAD (191 patients) and HUA with CAD (159 patients). Exclusion criteria were as follows: presence of renal impairment(CKD ≥ 3,urolithiasis, acute kidney injury, etc.), pulmonary edema, infectious disorders, current tumor and other severe illness; receipt of pro-uric acid excretory drugs(benzbromarone, probenecid or probenecid) within the previous week; receipt of hormone treatments therapy.

### Definition of clinical variables

HUA was defined according to the Chinese practice guideline for males of serum UA > 420 μmol/L [16]. CAD was defined as as the presence ≥ 50% stenosis in at least one of the major coronary arteries as indicated by coronary angiography [17]. The results of coronary angiography were assessed by two professional investigators using the Gensini score guide [18].

### Data collection

Sociodemographic information, such as age, and health factors such as smoking frequency and alcohol consumption were collected on hospitalization by questioning of participants.

### Laboratory analysis and sample preparation

Patients fasted for at least eight hours and 3 ml of venous blood was drawn from the elbow in the morning. Specimens were centrifuged with 3500 g for 5 min at least to isolate serum before experiment.

GDF-15 concentrations were determined by ELISA kit (R&D Systems, USA) by the color change of streptavidin-HRP, hydrogen peroxide and tetramethylaniline. The range of values detected by this assay was 0.78–50 pg/dL with intra- and inter-assay coefficient of variation 5% and 3%, respectively. Creatinine (Cr), uric acid (UA), urea(Urea),fasting plasma glucose (FPG), lipid profile parameters and non-esterified fatty acid (NEFA) were measured in serum by Advia 2400 (Siemens, Germany). Measurements were made by operators blinded to the patient’s condition.

### Statistical analysis

Student’s *t* test or Mann–Whitney U test was performed to analyzed continuous variables based on whether they were normally distributed or not and displayed as mean ± SD or interquartile range (IQR). The dichotomous variables were presented as percentages and compared by chi-square test.

To restricted cubic spline analysis, logistic regression was used and all risk factors were conducted in the restricted cubic spline analysis. To assess diagnostic capability of serum GDF-15/ NEFA concentrations and CAD model,the receiver operating characteristic (ROC) analysis was performed.

## Results

### Clinical and laboratory characteristics

Table 1 depicted the clinical and laboratory values for each subject. There were no discernible differences between the two groups in terms of clinical results like alcohol consumption, smoking, etc. Participants with HUA who also suffered from CAD had higher levels of Urea, Cr, TG, TC, UA, LDL-c, NEFA and GDF-15 while HDL-c levels were lower, suggesting compromised renal function and dysfunctional lipid metabolism.

**Table 1:** Clinical characteristics of HUA patients with and without CAD

**Characteristics**	**HUA without CAD (*n* = 191)**	**HUA with CAD (*n* = 159)**	*P*** value**
Clinical variables			
Age (year)	57 (52, 63)	59 (52, 67)	0.061
Diabetes Mellitus n (%)	21 (11.0%)	24 (15.1%)	0.254
Hypertension n(%)	59 (30.9%)	58 (36.5%)	0.270
Smoking n(%)	23 (12.0%)	23 (14.4%)	0.504
Alcohol consumption n(%)	16 (8.3%)	14 (8.8%)	0.887
SBP (mmHg)	131 (122, 145)	131 (123, 145)	0.639
DBP (mmHg)	85 (77, 96)	85 (77, 95)	0.850
Gensinin scores	3.0 (1.50, 3.50)	36.0(26.0, 62.0)	< 0.001
Laboratory variables			
Urea(mmol/L)	5.82 (4.74, 6.79)	6.20 (5.10, 8.25)	0.003
Cr (μmol/L)	79.0 (70.0, 92.0)	86.0 (74.0, 102.0)	0.002
UA (μmol/L)	504.0 (466.0, 536.5)	516 (486.0, 563.0)	< 0.001
FPG (mmol/L)	5.09 (4.61, 5.59)	5.00 (4.52, 5.90)	0.786
TC (mmol/L)	4.21 ± 0.05	4.52 ± 0.07	< 0.001
TG (mmol/L)	1.62 (1.05, 2.31)	1.85 (1.24, 2.47)	0.025
HDL-c (mmol/L)	0.97 (0.84, 1.10)	0.88 (0.76, 1.02)	< 0.001
LDL-c (mmol/L)	2.02 (1.55, 2.56)	2.31 (1.78, 2.83)	0.001
NEFA(mmol/L)	0.32 (0.24, 0.40)	0.45 (0.32, 0.60)	< 0.001
GDF-15(pg/dL)	5.78 (4.58, 8.57)	8.48 (6.67, 12.73)	< 0.001

*SBP* systolic blood pressure, *DBP* diastolic blood pressure, *Cr* creatinine, *UA* uric acid, *FPG* fasting plasma glucose, *TC* total cholesterol, *TG* triglyceride, *HDL-c* high density lipoprotein cholesterol, *LDL-c* low density lipoprotein cholesterol, *NEFA* non-esterified fatty acid, *GDF-15* growth differentiation factor-15

Normally distributed continuous variables are presented as mean ± SD, variables with skewed distributions are expressed as the interquartile range (IQR), and Categorical variables are presented as percentage (%).The independent t-test and Mann Whitney U test were used for comparison of continuous data, and chi-squared test was used for proportion.

#### Correlation of circulating GDF-15 and NEFA with UA

**Fig. 1:**
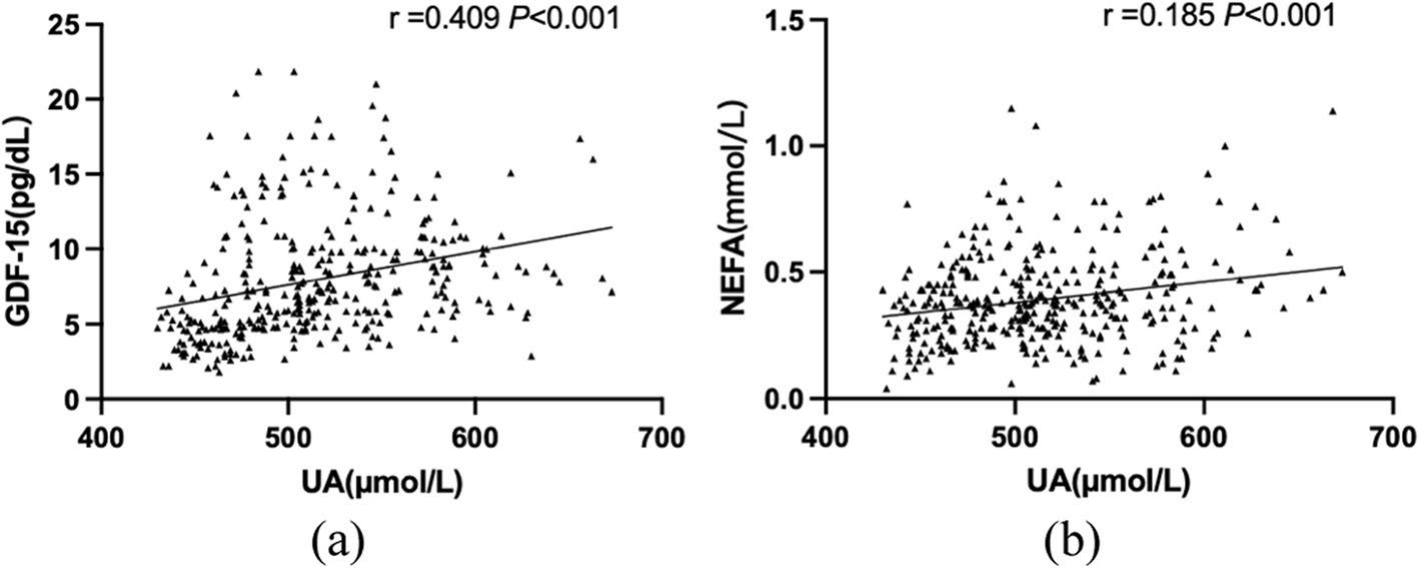
Correlations of plasma concentrations of GDF-15 (**a**)/NEFA (**b**) with UA of male patients with HUA

Associations between serum UA levels and concentrations of GDF-15 or NEFA in patients with hyperuricemia were tested using Spearman's correlation analysis. As shown in Fig. 1, GDF-15 concentrations correlated with UA (*r* = 0.409, *P* < 0.001, Fig. 1a), and NEFA concentrations weakly correlated with UA (*r* = 0.185, *P* < 0.001, Fig. 1b).

#### Logistic analysis of CAD prevalence in hyperuricemia patients

Serum GDF-15 and NEFA levels showed a correlation with CAD occurrence (Tables 2 and 3). Quartile ranges of GDF-15 and NEFA levels allowed the calculation of the OR for complicated CAD using the first quartile as reference. In crude model and after adjusting for clinical characteristics such as alcohol consumption, smoking, etc. (Model 1), serum GDF-15 levels correlated with CAD. Further adjustments allowed the construction of Model 2(adjusted for the same variables as Model 1 as well as Urea, Cr, UA,FPG, TC, TG, HDL-c and LDL-c) and the correlation remained statistically significant. The fully adjusted OR for CAD was 10.476 (95%CI: 4.158, 26.391). A similar tendency was shown for the correlation of serum NEFA with CAD prevalence and an adjusted OR of 11.244 (95%CI: 4.740, 26.669) was calculated.

**Table 2:** Association of CAD with serum GDF-15 in HUA men

GDF-15	n	Conc range,	OR(95%CI)		
**quartile**		**pg/dL**	**Crude**	**Model 1**	**Model 2**
Quartile 1	89	≤ 5.00	Reference	Reference	Reference
(low)					
Quartile 2	86	5.01–7.06	2.337	2.331	2.269
			(1.187, 4.601)	(1.178, 4.616)	(0.948, 5.433)
Quartile 3	88	7.07–9.80	4.320	4.182	4.128
			(2.221, 8402)	(2.130, 8.207)	(1.750, 9.734)
Quartile 4	87	≥ 9.81	10.354	10.455	10.476
(high)			(5.147, 20.829)	(5.100, 21.433)	(4.158, 26.391)
β			0.759	0.758	0.764
SE			0.110	0.113	0.148
*P* for trend			< 0.001	< 0.001	< 0.001

Serum GDF-15 was divided into quartiles (quartile 1: < 25th, quartile 2: 25–50th, quartile 3: 50–75th, quartile 4: > 75th percentile)

Crude: No adjustment

Model 1: Adjusted for age, smoking, alcohol consumption, Diabetes Mellitus, Hypertension, SBP, DBP

Model 2: Adjusted for the same variables as Model 1 as well as Urea, Cr, UA,FPG, TC, TG, HDL-c and LDL-c

**Table 3:** Association of CAD with serum NEFA in HUA men

NEFA	n	Conc range,	OR(95%CI)		
**quartile**		**mmol/L**	**Crude**	**Model 1**	**Model 2**
Quartile 1	80	≤ 0.27	Reference	Reference	Reference
(low)					
Quartile 2	88	0.28–0.37	0.967	0.988	1.188
			(0.521, 1.794)	(0.524, 1.861)	(0.553, 2.554)
Quartile 3	89	0.38–0.49	1.259	1.319	2.016
			(0.685, 2.314)	(0.706, 2.466)	(0.936, 4.342)
Quartile 4	93	≥ 0.50	8.667	10.148	11.244
(high)			(4.270, 17.590)	(4.879, 21.105)	(4.740, 26.669)
β			0.621	0.667	0.747
SE			0.106	0.110	0.135
*P* for trend			< 0.001	< 0.001	< 0.001

Serum NEFA was divided into quartiles (quartile 1: < 25th, quartile 2: 25–50th, quartile 3: 50–75th, quartile 4: > 75th percentile)

Crude: No adjustment

Model 1: Adjusted for age, smoking, alcohol consumption, Diabetes Mellitus, Hypertension, SBP, DBP

Model 2: Adjusted for the same variables as Model 1 as well as Urea, Cr,UA, FPG, TC, TG, HDL-c and LDL-c

#### Restricted cubic spline assessment of CAD incidence in hyperuricemia patients

**Fig. 2:**
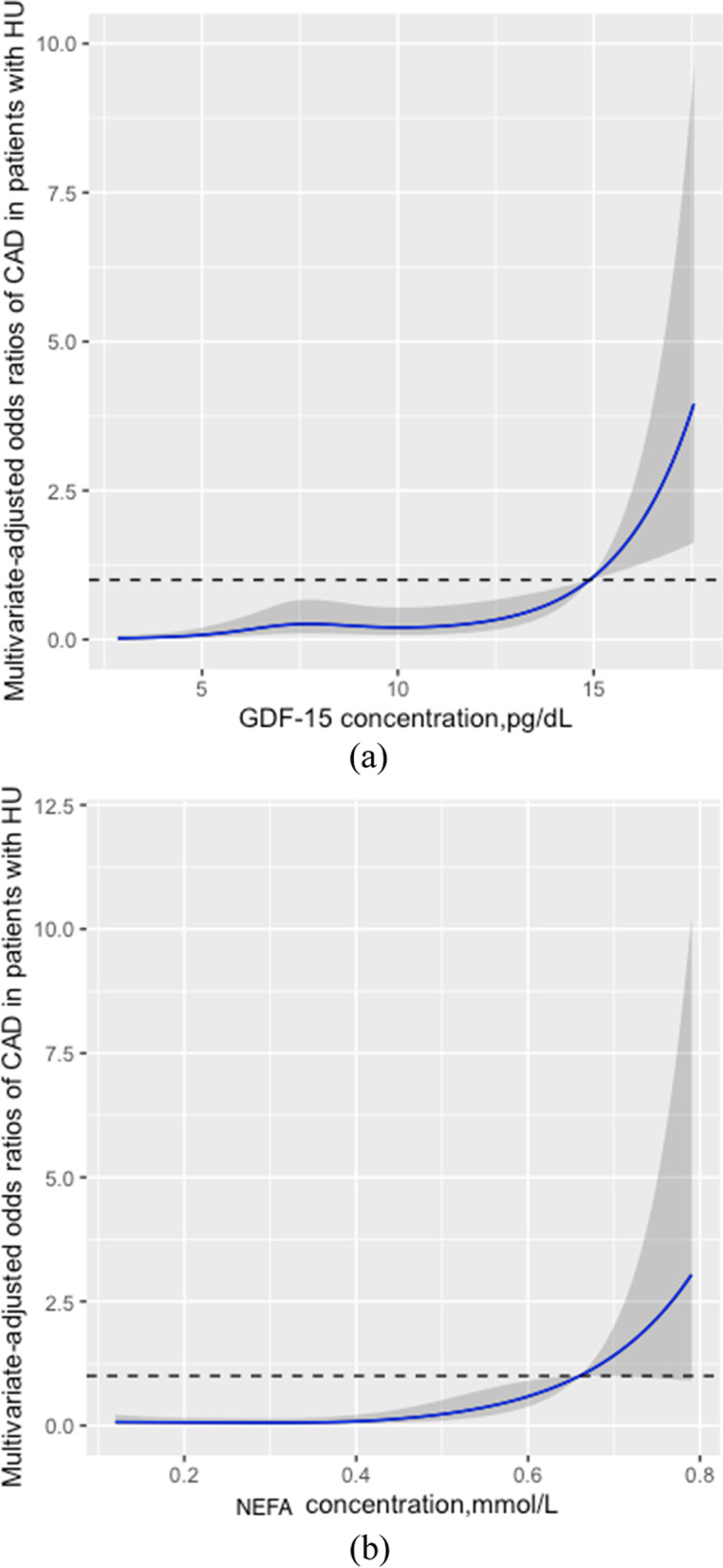
Restricted cubic spline model of the odds ratios of CAD. **a **Restricted cubic spline model of the odds ratios of CAD with serum GDF-15 in hyperuricemia patients. **b **Restricted cubic spline model of the odds ratios of CAD with serum NEFA in hyperuricemia patients. The dashed lines represent the 95% confidence intervals

Restricted cubic spline analysis of fully adjusted data showed a positive correlation between CAD prevalence and serum GDF-15 and NEFA (Fig. 2a and b).

#### Association of GDF-15 concentrations with CAD severity

**Fig. 3:**
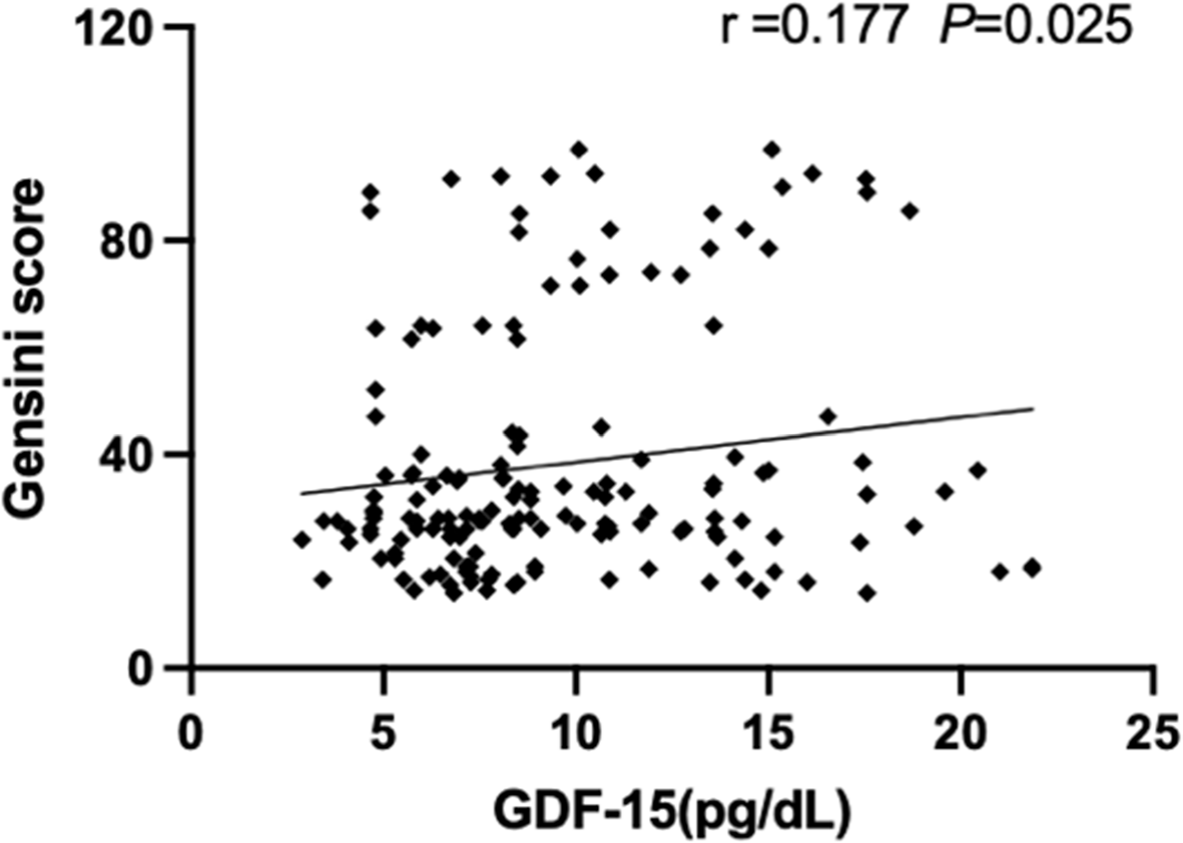
Correlations of plasma concentrations of GDF-15 with severity of coronary stenosis in hyperuricemia patients with CAD

In CAD patients, GDF-15 concentrations showed a weakly positive correlation with stenosis severity as defined by Gensini score (*r* = 0.177, *P* = 0.025; Fig. 3).

#### ROC analysis of predictive value of GDF-15 and NEFA

**Fig. 4:**
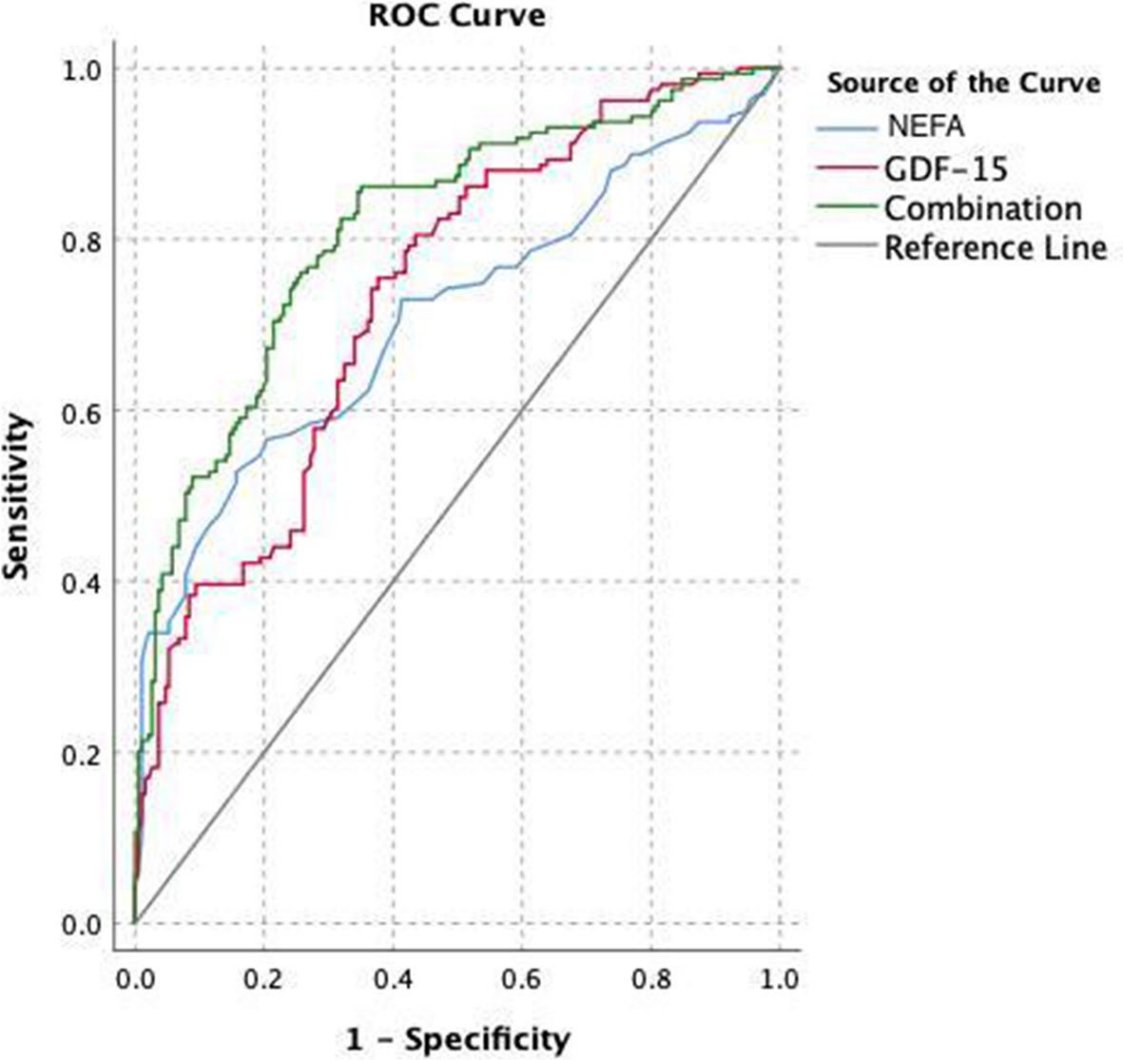
ROC curve of serum GDF-15 and NEFA levels in predicting the occurrence of CAD in hyperuricemia men

In Fig. 4, the AUC in predicting the incidence of CAD in individuals with HUA was 0.735. (confidence interval of 0.683–0.786) for serum GDF-15 with sensitivity and specificity of 75.5% and 62.3%, 0.378 Youden index. The AUC of NEFA for predicting patients with CAD was 0.709 (confidence interval of 0.653–0.765) with 52.8% sensitivity, 84.3% specificity and 0.371 Youden index. Furthermore, the AUC of the combination of GDF-15 and NEFA in forecast probability was 0.813 (confidence interval of 0.767–0.858).

## Discussion

The association between CAD and circulating GDF-15 or NEFA was assessed utilizing baseline data from 350 males with HUA. Serum GDF-15 and NEFA concentrations were positively correlated with CAD prevalence in males with HUA, a relationship which held after adjustment for potential confounders. The current report is the first to identify the combination of circulating GDF-15 and NEFA as a potential prognostic marker for CAD progression in males with HUA.

The correlation between risk of myocardial infarction or stroke with serum UA remained high even after other vascular-related risk factors were factored into the equation. High serum UA has been connected with various cardiovascular disorders [19], including higher mortality from acute myocardial infarction(AMI) in patients with excessive serum UA during retrospective observational studies [20]. A prospective cohort study analysis concluded that each 1 mg/dL increase in blood UA indicated a 20% increase in CAD and a 9% increase in all-cause mortality [21]. Cardiomyocytes are subject to dual anti- and pro-oxidant actions of UA. UA shields cells from oxidative stress and devotes more than half of plasma antioxidant capacity [22]. However, it also has pro-oxidant effects by decreasing endothelial nitric oxide generation, inhibiting vasodilation and stimulating the renin-angiotensin system (RAS) to enhance smooth muscle proliferation [23, 24]. Xanthine oxidase (XO) is thought to produce superoxides and hydrogen peroxide which stimulate LOX-1 lectin receptor generation to contribute to the LDL oxidation and deposition which stimulates atheromatous plaque formation [25]. HUA may make a multifactorial contribution to CAD onset with implications for diagnosis and treatment.

The connection between GDF-15 and various subtypes of CAD has been previously reported. Elevated GDF-15 has been associated to increased risk of death and detrimental occurrences after ACS in STEMI patients, confirming its potential as a biomarker for cardiovascular disease [26, 27]. GDF-15 shows minimal basal expression by many endothelial cells and macrophages but is greatly upregulated following tissue injury, inflammatory response and oxidative stress [28]. Macrophages respond to the pro-inflammatory macrophage colony stimulating factor (MCSF),IL-1 and IL-2 by producing GDF-15 in large quantities which then modulates the inflammatory response through apoptosis and IL-6-dependent vascular injury [29–31]. Circulating GDF-15 increased in individuals with AMI in correlation with inflammatory biomarkers [27, 32]. These pieces of evidence all indicate the relevance of GDF-15 for physiological processes of atherosclerotic lesions and CAD. Blood GDF-15 concentrations have been observed to spike quickly after cardiovascular injury due to heart failure, ischemiareperfusion and atherosclerosis [8, 33]. The current research detected that high GDF-15 concentrations were linked to high UA levels in all hyperuricemia participants, and GDF-15 levels were higher in male HUA patients suffering from CAD than in those with HUA alone, a finding that is consistent with previous work. Endoplasmic reticulum stress and oxidative stress are crucial contributors in the promotion of apoptosis, which has been linked to the pathophysiology of a variety of medical conditions, including cardiovascular disease. UA triggers oxidative stress and endoplasmic reticulum stress to induce endothelial dysfunction via the protein kinase C (PKC) pathway [34]. As a consequence of various intracellular organelle stresses (like mitochondrial stress or endoplasmic reticulum stress),GDF-15 expression can be increased in a variety of tissues and cell types to take part in a variety of pathophysiological processes [35, 36], such as heart failure, chronic kidney disease, pulmonary fibrosis, or cancer [37–40]. Additionally, there is mounting evidence that GDF-15 is involved in the pathophysiology of a number of metabolic disorders, including obesity-related insulin resistance and nonalcoholic fatty liver disease (NAFLD) [36, 41]. Besides, increased Gensini scores were likewise linked to higher GDF-15 concentrations, supporting the pan-vascular functions of GDF-15 that have been reported previously [42]. In light of this, GDF-15 appears to have promise as a valuable biomarker for detecting the presence of CAD in HUA patients.

NEFA are produced by lipolysis as an energy source for heart, liver and skeletal muscle and levels inform the clinical diagnosis of metabolic disorders. NEFA contribute to regulation of glucose metabolism, cell signaling and cell membrane formation and upregulate the expression of cytosolic XO to stimulate UA production [43–45]. Conversely, through altering the leptin-AMPK pathway, UA contributes to aberrant metabolic processes in adipose tissue, leading to increased blood NEFA and insulin resistance [46]. The findings of this study, which are corroborated by prior findings, revealed a slightly positive correlation between high UA and NEFA concentrations. CAD patients with HUA had higher serum NEFA levels, although the precise mechanisms whereby this situation arises remain unclear. Serum NEFA have been associated with increased CAD morbidity and poorer prognosis, including increased incidence of arrhythmias, AMI, all-cause mortality and sudden cardiac death [47–49]. NEFAs may also promote insulin resistance, accelerating the progression of obesity to type 2 diabetes [50]. Excess blood NEFA causes oxidative stress, inflammation and destruction of vascular endothelial cells, promoting rupture of atherosclerotic plaques and progression of cardiovascular disease [51–53]. The NEFA, linoleic acid, causes oxidative modification of LDL when oxidized, increasing atherosclerosis risk and contributing to CAD [54]. NEFA levels were higher in CAD after adjusting for covariates during the present study and may promote onset of CAD in hyperuricemic individuals.

Although it is widely known that HUA is an independent risk factor for the development of CAD, there is insufficient evidence to indicate that reducing UA levels in asymptomatic HUA individuals prevents CAD [55]. The utilization of Urate-lowering therapy(ULT) in patients with asymptomatic HUA is not recommended by the EULAR, the British College of Rheumatology, or the American College of Rheumatology [56]. Standard methods for CAD diagnosis present several limitations(for example, hs-CRP may reflect inflammation from a variety of causes, coronary angiography is invasive, high-sensitivity troponin is time-sensitive).As a result, sensitive biomarkers are needed to distinguish the presence of CAD in asymptomatic HUA patients. GDF-15 and NEFA are predicted to be such indicators.

### Study strengths and limitations

The current study enhances the understanding of preventative and treatment techniques for CAD patients. Blood GDF-15 and NEFA levels were discovered to be distinctive indicator of CAD in patients with HUA. measurements of serum GDF-15 and NEFAs are convenient and cost-effective and dynamic monitoring would enhance the grading and clinical evaluation of HUA individuals at high risk of cardiovascular conditions.

We acknowledge several deficiencies to the current investigation. Firstly, this was a small-scale study which may not apply equally to all racial or geographic groupings, giving a possibility of bias. Secondly, there are additional confounding variables, including drug history, that could also contribute to bias. Thirdly, long-term prognosis was not followed up and larger sample sizes are needed to provide confirmation of the current findings.

## Conclusions

Elevated serum GDF-15 and NEFA levels may be positively correlated with CAD prevalence in male HUA patients. Measurements may assist in the identification of high-risk individuals with CAD and prevention of adverse cardiovascular events.

## Data Availability

Data are not available to the general public owing to privacy considerations but will be made accessible by the author upon proper request.

## Abbreviations

GDF-15: Growth differentiation factor 15
SBP: Systolic blood pressure
DBP: Diastolic blood pressure
Cr: Creatinine
UA: Uric acid
FPG: Fasting plasma glucose
TC: Total cholesterol
TG: Triglycerides
LDL-c: Low-density lipoprotein cholesterol
HDL-c: High-density lipoprotein cholesterol
NEFA: Non-esterified fatty acid
CAD: Coronary artery disease
HUA: Hyperuricemia
